# Using Expertise to Improve through a Medieval Dutch Miscellany

**DOI:** 10.1093/shm/hkaf048

**Published:** 2025-08-31

**Authors:** Catrien Santing

**Affiliations:** Department of History, Faculty of Arts, University of Groningen, Oude Kijk in‘t Jatstraat 26, 9712 EK Groningen, the Netherlands

**Keywords:** miscellany, Hans Sloane, betterment, lifestyle rules, the good life

## Abstract

Based on the representative example of a miscellany (ca. 1500) of assorted advice texts from the Dutch town of Deventer, at present British Library Sloane 345, this case study argues that many late medieval urbanites strove to become masters of ‘the good life’, which functioned as part of a habitus of ‘betterment’. Scrutinising the many surviving informatory repositories like this one reveals signs not only of a developing domesticity and an increasing ‘vernacularization of medical knowledge’ during this period but also of a strong didactic streak relating to a human inclination towards (self-)enhancement. The Deventer miscellany was a tool for facilitating a long-term, experience-based ‘culture of expertise’ that emerged in the fifteenth century and resurfaced in the Enlightenment, providing knowledge which helped people to decode the world around them, understand their individual lives in connection to societal issues and find a path towards achieving a fulfilling existence.

In 1698, physician and president of the Royal Society, Sir Hans Sloane, purchased a voluminous miscellany produced in the Dutch Hanseatic town of Deventer. To date, the volume still bears the name of this avid collector.^1^ Produced around the year 1500, Sloane 345 contains a wide range of instructional materials. In addition to medical, predominantly lifestyle treatises in Latin and Dutch, the collection includes numerous prescriptions for medicinal unguents, and recipes for plasters, lotions, and hair dyes and the still-famous Deventer cake, alongside technical formulas for substances such as ink, paint and wax. At the end appears a liturgical calendar, which attests that this miscellany offers all-encompassing advice.^2^

The Deventer miscellany is not well known and has only been studied in the context of research on the history of surgery, pharmacy and other technical disciplines in the Middle Ages, as it contains what were at the time of its making popular treatises on these topics.^3^ The reason for this oversight is primarily the fairly standard character of its contents. To date, all over Europe, libraries store miscellanies or treatises with similar contents or subject matter to Sloane 345. This is particularly true of the Netherlands. The voluminous repertory of *artes* texts composed by Jansen-Sieben in 1979, for instance, lists many comparable miscellanies produced in towns in the vicinity of Deventer that are likewise understudied.^4^ Another reason for its neglect may be due to its ownership by the British Library, which received the enormous collection of Hans Sloane on his death in 1753, when his books became incorporated into the newly founded British Museum.^5^ The fact that half of its contents are written in (medieval) Dutch, that the codex contains standard texts of which many other copies are available and that it is rather plain and devoid of illumination, has also not done much for its fame.

Yet it is the unremarkable nature of the Deventer collection that makes it worth examining, as paying close attention to its contents—and potential ownership—reveals something fundamental about the usage of miscellanies in the Medieval and Early Modern period. At first glance, to the modern reader Sloane 345’s contents reinforce the prevailing image of the Middle Ages as an era of magic and mystery, but the following discussion proposes that for its original owners it would have offered guidance on an array of issues, from imminent death to a dysfunctional love life. As they addressed the concerns that medieval people faced throughout their lifetime, the many counselling miscellanies produced in the Late Middle Ages may be considered early examples of the then emerging genre of ‘how-to’ and ‘secret’ literature—that is, providing useful knowledge of real-world problems.^6^

However, this function should not be interpreted too literally. Sandra Cavallo has sharply observed that early modern medical advice books not only informed readers on the how but also on the why, making available to them not only a common body of knowledge but also an awareness of the rationale behind such knowledge; this applies just as much to the medical as to the non-medical advice in most manuscript miscellanies.^7^ In general, the information set out in miscellanies is well-organised, helping readers to cope with the wider world around them and providing them with tools that could make their lives better in an all-encompassing way. Sloane 345 can certainly be considered a representative example of such a collection, a comprehensive guide which helped its users to become experts in balancing their physical, mental and spiritual condition—not just offering practical knowledge but also the means to achieve betterment.^8^

Miscellanies of this sort were in common use by occupants of late medieval homes and their guidance was likely to have been supplemented by other books within reach, as well as through orally transferred expertise. To understand a miscellany’s function, then, we first need to understand the urban home in which it featured prominently. In recent studies on the late medieval household, several scholars have in this context identified a developing ‘domesticity’ during this period, along with the carving out of gender roles. Most significant in this regard is Luke Demaitre, who has positioned the many surviving obstetrical and gynaecological treatises from the Low Countries within the context of the ‘ordinary home’ built on the ‘domestic values’ of sexual harmony, enjoyment, comfort, decorum and a persistent striving for harmony.^9^ Yet, perhaps because his article focuses on Dutch medical texts, it has not been picked up by scholars, who have more recently worked on the particularities, values and mentalities of late medieval household culture, although his conclusions can be brought in line with their findings. Katherine French’s study of late medieval home life, rooted in historical household inventories, for instance, focuses on London as a locus of new ways of domestic behaviour and the start of an expanding consumer culture; in it, she assesses the behaviour, self-presentation and social expectations of the populace. French also identifies novel forms of civilian conduct in the late medieval period and expands its range to include the religious concept of the *vita activa*, which she defines as an ideology that ‘helped owners explain and justify their decisions with respect to their houses, possessions, management, and household behaviour’.^10^

Such attitudes can be linked to the Deventer miscellany in its capacity as a vademecum for betterment, a yearning towards a ‘good life’. This concept goes back to the reception of Aristotle in the Middle Ages via the Church Father Augustine, especially to the reworking of ideas on *eudaimonia* into the model of the good life by Thomas Aquinas.^11^ The ‘good life’ involved attaining personal fulfilment combined with societal perfection. The present article leaves such philosophical reasoning aside, aiming instead to demonstrate how the betterment practices that stemmed from this idea were widely embraced and helped people to learn how to become better persons, as we will see.^12^ Mastery of these practices in turn resulted in new modes of domestic and civic behaviour.

Taking this evolving approach to domesticity further, medieval guidance texts can also be connected to an emerging ‘burgeis’ identity, as sketched by Felicity Riddy. This mentality was expressed in what we can call ‘domestic geography’, i.e. the household space and its paraphernalia, but just as much in the promotion of industriousness and a shared language of the common good. In her description of the townsfolk’s habitus, Riddy points to the ‘rhetoric of settledness’ which was expressed in the mutual striving for comfort and welfare.^13^ David Harry, for his part, further elaborates on Riddy’s thesis to observe that in London, the coming into being of a civic community was steered by a language of ‘common profit’ in which moral edification, spiritual welfare and economic prosperity were interlinked.^14^

Such proactive attitudes can be found across medieval Europe, and the following proposes that Sloane 345 appeared at the onset of a burgeoning ‘culture of expertise’, a term coined by William Eamon on the basis of his longtime investigation into ‘secret’ literature. At the most basic level this refers to know-how that could be learned by doing and living through and by implementing instructions that were considered useful, making the reader more savvy.^15^ In their wide-reaching study of a plethora of advice books, Natasha Glaisyer and Sara Pennell fruitfully consider early modern didactic literature to be a medium that deployed a language of utility, seeing such texts as instruments of knowledge construction and communication.^16^

However, heeding Cavallo’s warning not to interpret ‘how-to’ texts in too reductive a manner, along with Glaisyer and Pennell I propose to understand such ‘expertise’ as something more than learning a skill and more like acquiring appropriate knowledge for attaining and practicing wisdom. Consequently, we can say that advice texts were potentially read constructively with an eye to achieving an active and meaningful life. To support this idea, the following discussion will examine the notions of expertise that run through Sloane 345, and which are reflected both in its ownership and how the miscellany was used. The active mindset was, in fact, a widespread phenomenon that existed outside the traditionally studied urban centres of England and Italy, but permeated the Northern Low Countries and probably the rest of Europe as well.^17^

Lately, the genre of ‘secret books’ has received substantial attention, but usually still places ‘secreta’ as mysterious and hermetically sealed.^18^ In contrast, Leong and Rankin have presented a compelling definition that connects ‘secret’ texts to knowledge texts in general, rather than treats them as a separate category. They state that books with ‘secret’ in the title are not hermetic, but instead ‘unlock the mysteries of nature, curing disease, maintaining good health, making practical every day, and even creating wondrous tricks. (…) Secrets, whether presented in the form of a printed tract or a gathering of loose handwritten leafs, encompassed a large genre and a wide range of actors’.^19^ This makes them fully part of, and not separate from, a wider shared culture of expertise that presaged later scientific paradigm changes. The existence of *Books of Secrets* point to a thirst for education that aimed to produce better human beings that dedicate themselves to raising the quality of society as a whole. These self-produced assemblages of various types of advice on life and death issues had become more popular among urban dwellers from as early as the fifteenth century. Such is proposed by Cavallo, who has shown that Early Modern printed medical advice books were founded in late medieval vernacular manuscript literature on the same topics.^20^

Because most secondary literature on advice books, and especially the printed ones, concentrates on the post-medieval era, when the genre of ‘how-to’ literature rapidly expanded, I propose to connect this research to the exploration of late medieval miscellanies. In doing so, I contend that medieval users of miscellanies were already fully integrated into a ‘culture of expertise’. The Deventer collection is merely one of the many examples of such a proactive attitude common at that time. If we are to understand informative miscellanies as manifestations of a rapidly increasing ‘culture of expertise’ in service of the search for a better life in the late medieval period, the understudied phenomenon of the miscellany as a tome containing an array of practical information has to be introduced and then connected to the emerging multifaceted genre of the more sophisticated ‘advice book’. This development will first be considered in the context of recent interpretations of such informatory texts in the historiography on domesticity, and of the desire for experiential and helpful knowledge. The main body of the article focuses on Sloane 345, presenting and analysing its contents in detail within the context of recent findings on expertise and betterment culture, and presents it as a characteristic example of an advice repository. Subsequently, the possible manufacture and ownership of the miscellany will briefly be considered in order to assess the aims of this typical advice volume. The conclusion will reiterate the main point that most miscellanies were a ‘toolbox’ for the creation of a good and meaningful life for non-medically trained citizens around 1500.

## What’s in a Name: The Miscellany

The hands-on, self-help approach of the composers and users of advice texts commonly resulted in self-assembled and sometimes even self-copied multi-text manuscripts like Sloane 345. In general, these are all classified as ‘miscellanies’, but the names ‘one-volume libraries’, ‘multi(ple)-text manuscripts’, ‘plural-text codices’, ‘Sammelbände’ or ‘composite volumes’ have also been proposed depending on the regional origin of the author. Despite strong criticisms of the marker ‘miscellany’, which some believe mistakenly brings to mind assemblages of a hodgepodge, happenstance character, this term is still the most commonly used. For this reason, it is employed in the current article, which targets a wide readership of social and cultural historians of health and does not limit itself to specialists of medieval manuscripts or incunabula.^21^

In terms of content, the study of miscellanies tends to pass over one vital aspect: namely the fact that such collections typically contain religious as well secular and technical material, which contemporaries would have regarded and exploited as complementary and cross-pollinating.^22^ In recent years, scholars have rightly drawn attention to numerous collections that were at the service of religious guidance and the achievement of religious experience. Indeed, during the fourteenth and fifteenth centuries, compilations entitled *florilegia* or *rapiaria* were produced for the purpose of providing mental and spiritual relief. They often comprised personal reading notes—as was, for instance, the case in collections of the Modern Devouts, the spiritual movement originating in Deventer, the hometown of Sloane 345.^23^ This finding is important, because it departs from the previous emphasis on either the secular or the spiritual content of miscellanies, especially those containing information on medicine and pharmaceutical knowledge. Thus, while there may be differences in composition and emphasis, I suggest not making a distinction between religious and secular collections. That such a distinction is not fruitful becomes evident from the various Dutch miscellanies listed in Jansen-Sieben’s *Repertorium* and is crucial to the issue of the Deventer miscellany’s original production, composition, ownership and function, as we will see below.

At the time of the miscellany’s making, individuals were willing to go to great lengths to make them; in many cases, they copied them themselves or spent considerable amounts of money on purchasing and adapting instructions relevant to them. As such they reveal the existence of what has been coined ‘pragmatic’ or ‘utilitarian’ literacy which became common among town-dwellers of the fifteenth century.^24^ Therefore, it is wise to build interpretations of miscellanies on complete surveys of their various but interconnected components, i.e. the ‘whole book’, meaning the written and printed texts, their outlay, order and execution, including the paratexts.^25^ Sloane 345 seems to be such a ‘bespoke’ urban product, comprising a variety of transcriptions of common information texts taken from Antiquity and the medieval period supported by a helpful layout and makeup. This means that a citizen’s strategies for improvement might be gleaned from an in-depth study of a manuscript codex containing an array of guidance texts.

To give an early example: the *Régime du Corps* (1256), originally authored by Aldobrandino of Siena—which was also purchased by Hans Sloane—shows clear signs of this hands-on approach. Originally, the treatise was written for the French court. Having received courtly acclaim, it was revised and tailored to the needs of a rich Florentine businessman, Bernard de Florenche, to whom the Sloane copy is dedicated.^26^ Quite exceptionally for its type, the manuscript is splendidly illustrated with pictures that highlight the practical nature of the advice. The overall tone is one of business coupled with pleasure: the sexual act, for example, is vividly portrayed. In similar fashion, the merchant who purchased it was also given the opportunity to gain useful knowledge about the complexities of seasonal clothing (Figure 1). A telling detail is the fact that this copy of Aldobrandino’s *Régime* is bound together with another exquisitely illustrated didactic treatise, Gautier of Metz’s 1245 *Image du monde*, which is an encyclopaedic work about the Earth and the universe and their creation and configuration, and thus instructs its readers on how to interpret the Creation.^27^ This means that Bernard de Florenche at one time commissioned a showpiece manuscript volume consisting of two texts that jointly display the habitus of the well-to-do man of the world. Its functional content and useful approach to well-being also reverberate throughout the more prosaic and plain multi-text codex Sloane 345.

**Fig. 1:**
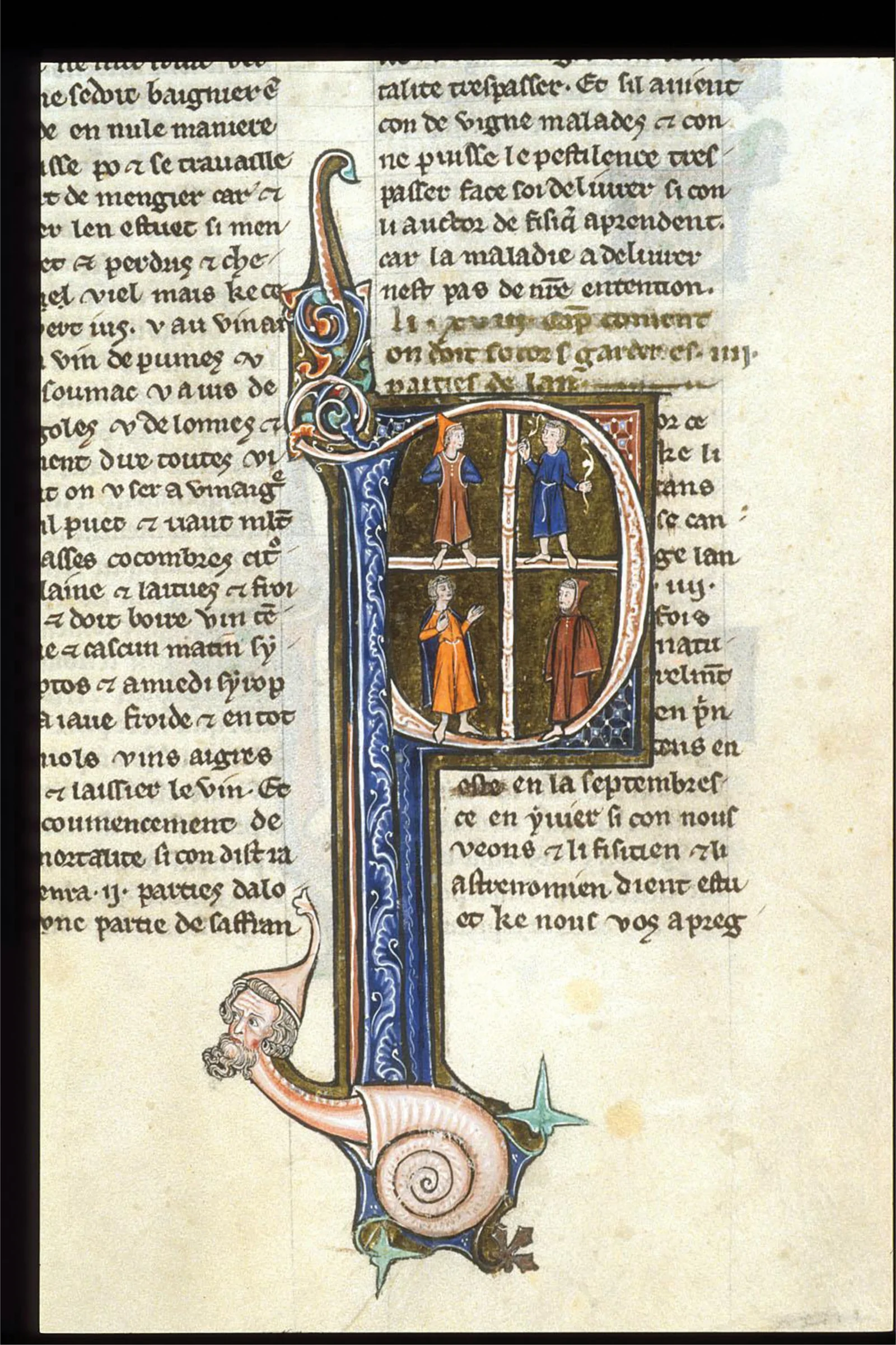
Aldobrandino da Siena, *Régime du Corps*, Seasonal Clothing, British Library Sloane 2435, c. 1285, f. 23. Courtesy of British Library

The varied content of most miscellanies and their popularity among medieval households across Europe attest to another aspect of ‘betterment’, i.e. that the urge to educate oneself was broad—in geographical, disciplinary, linguistic and chronological terms. Yet to date, contemporary attempts to improve mental and physical well-being and living conditions remain underexplored. Those historians who do acknowledge the existence of a desire for healthy living and betterment, including Rudolph Bell, Sandra Cavallo, Tessa Storey and Paul Slack, tend to place it rather late and after the Middle Ages. They also seem to overvalue Italian and English influences on this social development.^28^ Nonetheless it is exactly this recent scholarship that allows us to redress the balance and conceptualise the late medieval mentality as seeking edification and cherishing the principles of common profit.^29^

The proliferation of rich encyclopaedic advice texts, especially those incorporated in miscellanies and covering a range of topics, including statecraft, ethics, religion, physiognomy, astrology, alchemy, magic and medicine (especially gynaecology), flourished throughout Europe from the central Middle Ages onwards. All sorts of handwritten instructions, including individual and collective well-being advice, found a growing market amongst the inhabitants of the busy late medieval towns of north-western Europe, which saw an increase in (pragmatic) literacy.^30^ A new category of lay readers appeared, which is to say—and the categories should not be applied too rigorously—non-clergy, non- or partly university educated and non-medical professionals, for whom assorted guidebooks were authored and published. Because of the brevity and practicality of these books, physicians, surgeons and apothecaries occasionally may have consulted them; but an emerging public readership was to be found ‘beyond the professions’, as Sabrina Minuzzi has convincingly demonstrated.^31^

A clearer picture of the general usage of such late medieval forms of guidebooks that ‘offered help’ emerges when taking a closer look at the array of counselling texts known as *speculum* or *regimen*. They appear in numerous miscellanies, albeit often in an abridged, translated or otherwise customised form. The two rubrics developed in the Central Middle Ages and both denote instruction manuals. Although the first tends towards an encyclopaedic comprehension of rather lofty topics, while the second concentrates on lifestyle regimes, there is considerable overlap in terms of subject matter, set-up and aims. Some guides were focused on particular categories, whereas others were aimed at specific target groups and offered tailored advice.^32^ Lifestyle guides were compiled for pilgrims, scholars, the elderly, monks and clergy, (pregnant) women, housewives and others. During the fourteenth and fifteenth centuries, other rubrics came into fashion, such as *Secreta* (secret), *Problema* (problem or issue), *Tacuinum* (table), *Hortus* (garden), *Imago* (picture) or vernacular equivalents of these, such as *Castle*, *Treasure*, *Calendar* and *Almanack*. These often entailed short and rather sketchy overviews of their subject matter.

Given the abundance, and, thanks to the rapid adoption of the printing press, further proliferation of advice texts on well-being, the inclination in modern scholarship to categorise these sources is understandable. However, over the last decades, scholars have notably struggled to classify information repositories and instead invented labels such as ‘household books’, ‘family or husbandry books’ and ‘commonplace books’, or have employed the medieval term *secreta*, as in ‘secret books’, as discussed above.^33^ Yet, all of the miscellanies can truly be thought of as ‘practical books’ in the sense of useful and helpful. They were utilised within the sphere of the household and would have served for a very long time as reference points for resolving all sorts of issues.^34^ Nor should these labels be restricted to sixteenth century and/or to printed materials; it is important to note that the kind of information these manuscripts contain had typically been part of multi-text codices for centuries. Elaine Leong and Alisha Rankin, as well as Sandra Cavallo, have extensively demonstrated that we should make no radical distinction between manuscript and printed advice literature.^35^ With unremitting zeal, male and female, lay and religious householders continued to consistently copy for themselves recipes, household tips and advice texts, storing them in their libraries next to printed bibles, collections of saints’ lives or illustrated herbals.

The scholarly thinking on domesticity, the common good and expertise discussed so far in this section suggests that this comprehensive didactic literature was prevalent in the late Middle Ages and widely available outside Italy and England, the two best-studied regions. And while the term ‘secret literature’ is used to refer to hands-on medical, pharmaceutical and technical advice on the European continent, in the Low Countries, this type of manual is usually termed *artes* literature and in Germany known as *Fachprosa.* The terms refer to informative texts aimed at the transfer of knowledge for improving expertise, i.e. not just narrow professional ‘learning’ but also wisdom and thus moral betterment, as exemplified by Sloane 345.^36^ Yet, scholars of Dutch guidance literature have disregarded these miscellanies’ textual and material context, mainly restricting themselves to linking single texts to their authors and thus establishing their written and printed textual traditions.^37^ With regard to the Deventer miscellany, this is understandable given that much of the content can be traced back to the famous *Regimen of Salerno*, and because it includes basic surgical and gynaecological knowledge as well as pharmaceutical and technical formulas. However, the miscellany’s unexceptional nature makes it even more interesting for probing the culture of expertise and the possible Europe-wide urge for betterment.

## Sloane 345 as an Information Repository

In this section, Sloane 345 will serve as a representative case study of typical advice literature. The aim is to determine how diverse topics—from medical and gynaecological, to pharmaceutical, culinary and technical, to more general and also religious advice—were all related to the creation of a good life in the context of the urban community and were aligned with the aforementioned characterisation of advice texts as a whole. The online manuscript catalogue of the British Library provides a clear listing of its contents, but it is equally important to examine the miscellany as a ‘whole book’, thus interconnecting its assorted contents in order to get a sense of the aim and function of this and other miscellanies like it in late medieval Europe.^38^

The many surveys, abstracts and lists that it contains reveal the composer’s practical and profit-seeking approach. There is a glossary of surgical terms and illnesses (ff. 37r-38v and 83v-85r), in addition to a list of Latin botanical names with their Middle Dutch equivalents (f. 83v). Although the collection seems to have been a work in progress, as the order of topics lacks an index or table of contents, its layout is helpful and clearly arranged (Figure 2). There are initials in red, brown and blue, underscorings, small initials next to paraphs and rubrics in red, while in the case of alphabetisation, capitals in the margin show the reader where he or she has landed. Other indicative signs and roman numerals help those consulting the manuscript to find their way and retrieve the proper information. Being no showpiece—it contains no pictures—the hand, or very probably hands, become more and more sloppy towards the end. It is also telling that the codex is made from paper, not parchment, which points to its practical everyday use and the likelihood that it lay within constant reach of those in need of counsel.

**Fig. 2:**
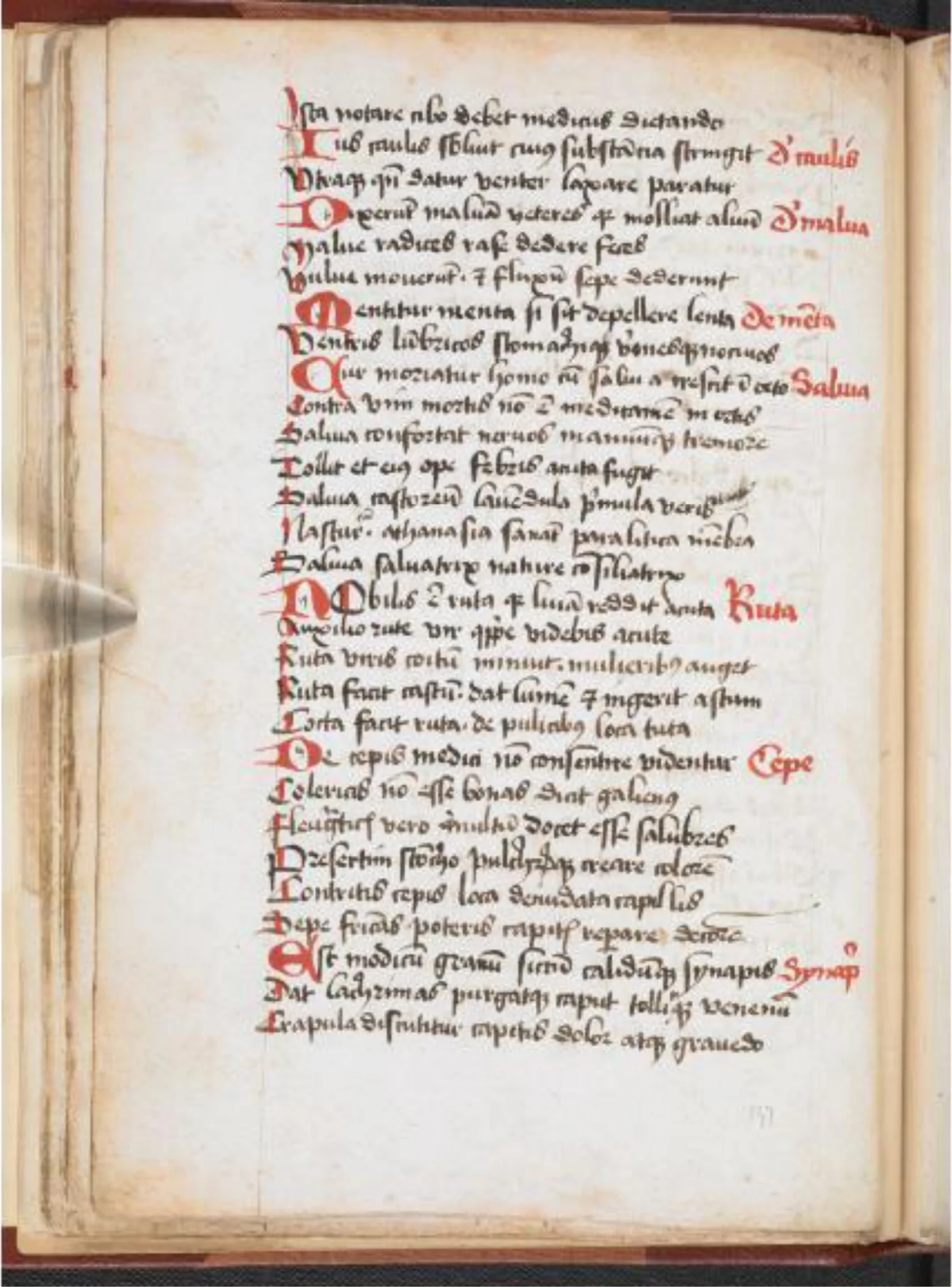
BL Sloane 345, f 12r. Page from the *Regimen Sanitatis Salernitanum.* Courtesy of British Library.

The common denominator of the materials in this collection is that they provide information on overall well-being, but the contents highlight health matters in particular, mainly concerning lifestyle. In fact, the compilation contains three *regimina sanitatis*. The first is a personalised health compendium for a bishop of Utrecht, David of Burgundy (1426–1494), authored by one of the Deventer town physicians, Reyner Oosterhusen (ff. 1r–6r). During his tenure, Bishop David also acted as the secular lord of the town of Deventer and in this capacity visited the rich Hanseatic town various times^39^ (Figure 3). It is known that he suffered from poor health, especially from gout—at the time a typically elite malady.^40^ The initial intention of the writing of the text seems to have been to secure a court appointment or to become somehow financially remunerated. Maybe for this reason, the *regimen* is accompanied by a proper appraisal of the medical profession and the requirements to practice it, credited to a Johannes Behemens (f. 7r).^41^ Since both Bishop David and Oosterhusen had died by the time of the miscellany’s manufacture, a citizen-owner must have recognised the prescribed regimen’s value for ensuring his or her own well-being and therefore copied it. The fact that the regimen in the miscellany had initially been drawn up for a prince, the (illegitimate) son of Duke Philip the Good of Burgundy, would have worked in its favour, similar to the situation with Arnald of Villanova and his version of the famous *Regimen of Salerno*, dedicated to the king of England, James II.^42^

**Fig. 3:**
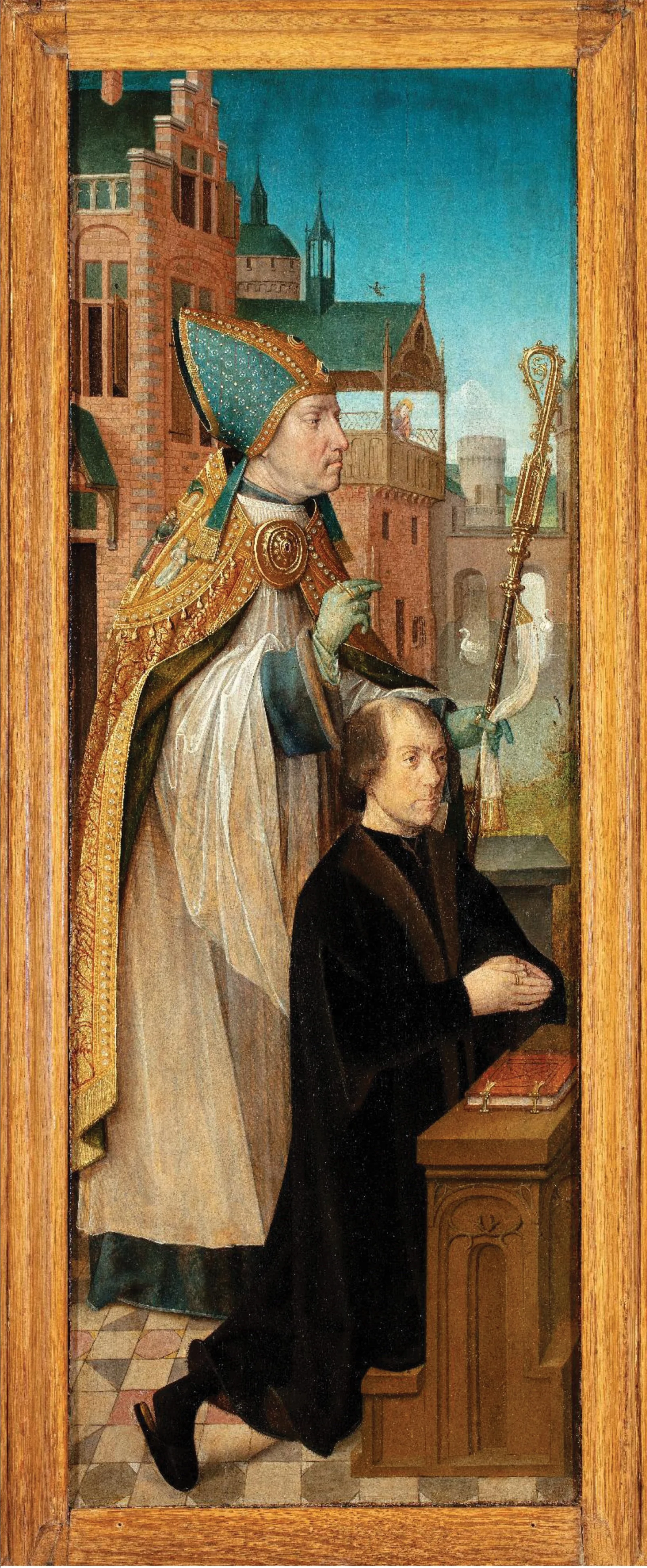
Bishop David of Burgundy as Saint Martin. Left panel of Triptych painted by the Master of Delft, ca. 1500. Rijksmuseum Amsterdam. Creative Commons.

The latter *regimen sanitatis* was the basis for all later medieval regimens, and in the miscellany it immediately follows the lifestyle advice for Bishop David. The title *Flos Medicinae*—Flower of Medicine—points to the version authored by Arnald (ff. 8v–14v). A third lifestyle rule is included, which is a concise and lapidary schedule for the entire year named *Regimen corporis per circulum anni*, which gives advice in the style of the famous *Tacuinum sanitatis* (ff. 144v–145v). As a matter of fact, in many medieval manuscripts, it also happens to accompany the Salerno regimen.^43^ Moreover, there are other short texts in the same dietary mode, i.e. general observations providing essential information for leading a balanced lifestyle, including advice on the four complexions and the influence of fasting, along with information on venesection, and, for instance, on the impact of the various winds (f. 144r).

A similar illuminating approach is found in a series of printed natural-philosophical problems in the form of questions and answers that in the Middle Ages were attributed to Aristotle. These *Questiones* appear in the miscellany, combined with others that were probably authored at the Medical Faculty of Salerno (ff. 138r–141). Both texts were widespread, partly because of the explanations they give of biological phenomena that might have stirred the imagination of their readers. We find narrated myths about bears—such as that they lick their young into shape after birth—and learn that beavers are called *castor* because they castrate themselves when in danger, stories which reflect an eager desire to penetrate the wonders of the Book of Nature.^44^

Less obvious examples of texts intended to serve the cause of household profit are medical therapeutical treatises scattered throughout the manuscript. Three of these are attributed to Johannes de Ketham: *Chirurgia parva* (ff. 118r–127v), *Liber de matrice mulieris* (ff. 128r–130r) and *De diversibus infirmatibus et remediis earundem* (ff. 131r–136v). At the time, these very common medical works were already in print, often found together under the title *Fasciculus Medicinae*, and soon they even appeared in the vernacular.^45^ Possibly Ketham’s printed *Fasciculus* was available in Deventer and had been copied out of curiosity.

Next to these substantial medical works, the manuscript contains short medical observations by Isidore of Seville, Constantine the African, Richardus Anglicanus and Pope Innocent III, presented together as *Tractatulus de practica medicina* (ff. 66r–70r). It should be noted that current standard medical treatises, such as the works of Hippocrates, Galen, Avicenna and other Arab physicians, are absent. They may have been too much a part of the university medical curriculum to make it into miscellanies intended for the general public. This does not fully apply to (Ps)Hippocrates’ *De signis mortis* (ff. 20–21r), a non-specialist short treatise on the signs of approaching death, which would have provided very useful information for those dwelling in towns, which were frequently stricken by epidemics.

The same urban environments, which experienced frequent accidents in workshops, uprisings and military expeditions, might also have benefitted from the well-known treatise on wounds written by the young Lanfranc of Milan in Dutch (ff. 70r–78v), which is also copied in the miscellany.^46^ This treatise is followed by several recipes on the healing of wounds, such as what to do when suffering a blow to the head and getting a bump from it, which are recommended in an addendum (ff. 142–143v).^47^ In fact, numerous surgical treatises were translated into Dutch during the Late Middle Ages and are still found in great numbers, suggesting that they met a wide demand for knowledge on the topic.

In his article on the popularity of texts on the ‘special secrets’ of women (*Secreta mulierum*), Luke Demaitre emphasises the wealth of gynaecological and obstetrical advice available in late medieval Dutch cities.^48^ The rich information on these matters found in the Deventer miscellany more or less echoes his findings.^49^ Alongside Ketham’s well-known *Liber de matrice mulieris et impregnatione* (Book on the female uterus and pregnancy), the codex contains copious guidance for women.^50^ Although Ketham’s gynaecological survey was most likely copied because it was part of his comprehensive *Fasciculus*, its inclusion does also reflect the curiosity about human conception and reproduction observed throughout Europe at the time.^51^ There are no other full treatises on female issues transcribed in the manuscript, but we find many formulas for concoctions that claim to stimulate menstruation or to purge the female reproductive organs.^52^ Some of these recipes promise to untie the foetus from the womb. The use of particular vermifuges is also considered to have the same effect. In both cases, the danger of abortion is mentioned only in passing, almost casually, seemingly without any prejudice let alone religious warnings. One gets the impression that these remedies were widely known and may have been used as birth control.^53^ In addition to these, there are instructions on how to conceive a son or daughter, how to induce a mother’s milk and how to cure sores on the breast as well as to reduce inflammation of the nipples.^54^ A remedy for helping a mother thought to be bewitched to produce milk belongs to the same obstetrical category.^55^ The common factor connecting all this type of information is the aim to make the lives of women and girls easier, safer and more comfortable.

This compassion shown towards women and their specific problems contrasts sharply with folio 141r/v, which contains a discussion of the ‘female’ as a peculiar phenomenon. These passages betray a deeply ingrained misogyny which is often encountered in satirical texts that circulated in monasteries throughout the Middle Ages. In Sloane 345, these statements are attributed to Isidore of Seville, Augustine of Hippo and Vincent of Beauvais. The latter dubbed women an ‘incorrigible animal, the confusion of man, an impediment to chastity, a stinking rose’. In English, the same verdict is pronounced in Chaucer’s ‘The Nun’s Priest’s Tale’, in which Chauntecleer silences Pertelote, one of his seven wives, after her explanation that dreams are the result of disordered humours. The cockerel snaps at her: ‘Mulier est hominis confusio’—the woman is the confusion of man.^56^ However sexist these warnings may now seem, at the time they were rather mainstream. Like the recipes for easing menstrual pain, late medieval urbanites may also have found these experiences of their predecessors valuable for navigating the practicalities of life.

The rest of the information in the Deventer miscellany is clearly designed to assist in coping with human existence in an even more down-to-earth respect. Recipes in Dutch concerning all kinds of medicinal and culinary potions and substances are to be found at various locations in the manuscript; by and large the information is well-organised and supported by an alphabetical list. Much of the information concerns the healing of wounds, eye irritations and skin problems, which were all prevalent in the late medieval city. There are also numerous recipes for cooking and baking, especially luxury confectionery such as cakes, cookies, sweet roses and candied orange peel, as well as syrups. One can, for instance, find out how to prepare brandies, among which there is a very early appearance of *brandewijn* or Dutch brandy (ff. 51r–v).^57^ The distinction between the culinary and the pharmaceutical is therefore not very strict, a feature common to late medieval *artes* collections. The same holds true for two contemporary popular treatises attributed to Arnald of Villanova. The first concerns the medicinal use of the oak tree (ff. 15r–16r). A second sets out details for the production and culinary and medicinal consumption of wine-based remedies (ff. 16v–20r). All these products will have added zest to the urban life style.

Nonetheless, the majority of the provided preparations deal with technical and artisanal matters concerning livelihood and the creation of comfort. Instructions are given on how to prepare ivory, soften and repair paving bricks, clean silk and chamois, and produce wax for seals in the right colours.^58^ Extremely interesting is a series of recipes for paint and varnish, which are in Dutch despite the Latin heading *De diuersis coloribus picturis et tincturis* (ff. 23r-34v).^59^ Since many of these formulas relate to the manufacture of gold and silver paint, as well as various colours of ink that were all used for text copying, illuminating and letter writing, they seem to have been collected with an eye to book production, for which the town of Deventer was famous at the time.^60^ In addition, retable painters and those who had to colour fabric or metal would have found ample guidance in selecting the right components and employing the correct production methods in the treatise. Regarding the fabrication of luxury goods, the recipes tend to suggest less expensive alternatives to genuine gold and silver that circumvent their use. It is, for instance, more than once explained how copper, lead and silver can convincingly pass for gold.^61^ This seems to have been an important issue, because towards the end of the miscellany it is also described how rings made of copper could be upgraded to look like silver or gold. Instructions on how to create a window out of paper instead of costly glass, and how to correct bad wine or beer that has gone sour, are also given.^62^ One may conclude that this type of advice was offered at particular points to citizens who every now and then struggled financially and sought to maintain appearances.

This desire to keep up appearances also holds true for the numerous skin lotion formulas in the miscellany. In part, they claim to heal the many skin problems of the time—such as pimples, freckles and sores—but most were undoubtedly intended to beautify the face. Spots, freckles and redness should be whitened, for instance. And while many of these remedies and most of their ingredients were quite common, more specialist, pricy potions are recommended to fight the signs of old age.^63^ One of them advertises the use of precious metals soaked in the urine of a virgin and cooked in the breast milk of a mother nursing a son; the concoction must then be mixed with red wine and eggs.^64^ The resulting potion is touted as a universal wonder drug, promised to cure leprosy, clear the skin of any flaws and heal the eyes. Elsewhere in the manuscript, the origin of this elixir for eternal beauty is attributed to Aristotle, who allegedly once prescribed it to Calistris, queen of the Amazons.^65^ This is a typical case of a treatment reputed to be highly effective because a famous authority once prescribed it to a VIP.

Though the core business of the shorter recipes involves cooking, treating physical ailments and producing technical products, there are also medications aimed at securing mental balance, treating folly—especially errors of the heart—and restoring human happiness by comforting the brain.^66^ Such guidance often hinges on creating calm sleep; in particular, talking while asleep should be avoided. Nonetheless, *litaergia*, that is, too much sleeping and not enough working, was considered harmful to stability as well.^67^

Now and then, magic and religion merge in the formulas of the prescriptions, but on the whole neither figure prominently in the collection. Religion is typically disguised, such as in a recipe for the preparation of the founding of sheet metal. In this case, it functions to determine the time needed for mixing the ingredients. To acquire the perfect end product, it is prescribed to put a slice of rye bread on a stick and stir for the duration of a ‘miserere mei’.^68^ These Latin words refer to (Vulgate) Psalm 50, which is a penitential sung by King David. It is unlikely that the litany merely functioned as the equivalent of a clock or hour-glass: medieval artisans were supposed to say their prayers during work, and this seems a good example of killing two birds with one stone. By consciously stirring a concoction at work while reciting a psalm, one could maintain both mental balance and spiritual well-being.

While the weight of Sloane 345 tilts towards guaranteeing physical and material comfort, the very end of the codex contains both a calendar of movable feasts and the aforementioned *Regimen corporis per circulum anni* (ff. 144r–145v), being a regimen in almanac form. The two pieces should be interpreted as a diptych for integrating spiritual and physical well-being, in the sense that one half covers the crucial points in time marking the progress of the soul through the liturgical year while the other provides guidance for the maintenance of daily good physical health throughout the seasons. This speaks to the late medieval belief that a good Christian must strive to lead a responsible and successful religious life for which sound physical health was a precondition.

Just like many other miscellanies, Deventer initially included printed advice, which was bound together with handwritten instructions. Originally, there were three printed works enclosed, which later became detached from the original miscellany. Regrettably, they can no longer be located among the several copies of the works owned by the British Library. The first printed text was between folia 130 and 131 and entitled *Problemata Arestotelis determinantia multa questiones.* It concerns a collection of natural philosophy problems written in a question-and-answer format which in the Middle Ages was thought to have been the work of Aristotle, as explained above. In the original codex, the booklet was fleshed out with a selection of 27, also pseudo-Aristotelian, *Salernitan Questions* (ff. 138r–141r).^69^ The two other printed instructions that were once part of the Deventer miscellany were bound at the very end, being works on abacus-based accounting using *rekenpenningen* or monetary coins. One was the *Algorithmus linealis cum pulchris conditionibus* by Balthasar Licht (Leipzig 1500), the other a similar brochure by Johannes Huswirt entitled *Enchiridion novus algorismi* (Cologne 1501).^70^ Whilst the scope of the printed matter is somewhat different than the lifestyle guidance of the manuscript parts, in practicality and applicability they would have conformed just as well to the betterment needs in terms of welfare of any inhabitant of a flourishing late medieval trading town.

The dual language usage of the miscellany is worth some extra consideration because it does have a bearing on the audience. The most common medical information in Sloane 345 is phrased in Latin, and this also goes for the texts on natural philosophy and arithmetics. These disciplines were traditionally taught at the universities and therefore recorded in a classical language. This does not, of course, apply to the information on surgery, which for the most part was practised by professionals trained in guilds and thus was usually written in the vernacular. As the surgeons became increasingly well-educated, however, their guides reveal a level of bilingualism, as is the case in the Deventer miscellany. Nevertheless, whether written in Latin or in the vernacular, all parts are phrased in an accessible style and wording, suggesting that they could have been read and understood by citizens with some education who wanted to expand their expertise.^71^

The above survey has revealed the breadth and wealth of information to which the owners of miscellanies had access. Sloane 345 can be deemed a serviceable manual of well-being and welfare typical of fifteenth- and early sixteenth-century miscellanies, as many contain *regimina*, provide culinary and pharmaceutical recipes, and habitually give lists of herbs combined with the occasional prayer and magic formula. The Deventer miscellany also shares many characteristics with household and husbandry books, and likewise with other kinds of advice texts, such as secret books. The very fact that the collection is so unexceptional in form and content—the Netherlands alone produced ca. 1000 of these that are still extant^72^—gives evidence of how widespread this culture of betterment was.

## Ownership and Use

The catalogue of the British Library states that Hans Sloane bought the Deventer miscellany out of the inheritance of Francis Bernard (1628–1698), one-time apothecary and physician to King James II.^73^ Like Sloane, Bernard was interested in medical practices from the past, such as those set down in medieval manuscripts. The ownership of the miscellany—who ordered and/or copied this storehouse of advice texts, and with what intention—should give us a clue as to its original function. To address all of these questions, a systematic comparison with other miscellaneous collections from the time and region would be required, which unfortunately goes beyond the scope of this article as an in-depth case study. To somewhat compensate for this deficiency, and also to highlight the promising possibilities of ‘whole book style’ research on late medieval miscellanies, comparisons with a few other manuscripts from the same region will be made that display the same orientation towards betterment or the creation of a Good Life.

Given the nature of Bernard and Sloane as collectors, one might guess that the miscellany was once owned by a local physician. The town physician Oesterhusen, the author of one of the *Regimina*, is such a candidate; another is one of his colleagues, as the town of Deventer over the decades had employed various town physicians and surgeons.^74^ Since recipes make up a large part of the content, the volume could also have been in the possession of a well-educated apothecary or other seller of herbs, spices and ingredients for the making of specialist substances.^75^ Such a professional would have prepared medicinal prescriptions as well as sell ingredients for technical and cosmetic preparations, and thus would have been expected to advise buyers on administering them. At the time, apothecaries often had some medical training: some even attended university, so it can be presumed that they were able to comprehend the Latin in the texts and recipes.^76^ The lists containing ingredients, including the Dutch translations, would also have been of great use to owners of a pharmacy.

The paratext of Sloane 345 also points to functioning beyond the professions of doctor, surgeon, and apothecary. This is especially the case in the transcript of the *Regimen of Salerno*, where the texts of the prescriptions and commonly applied advice on the non-naturals, the external influences on health, happen to have red headings accompanying glosses with equivalent wordings and other instruments for clarification, whereas the lengthy descriptions of how and when to bleed lack such aids and commentaries. A non-professional owner might have been content with a basic comprehension of phlebotomy, for instance: for actual bleeding, the owner would have turned to a barber or surgeon. This, of course, does not exclude the possibility that it was used by a physician or apothecary, as the tips and tricks might have come in handy to them; Sabrina Minuzzi, in fact, notes that many medical texts, especially handwritten summaries, regimens and *materia medica*, functioned at the intersection of professional and popular usage.^77^

Therefore, a well-educated bourgeois owner with broad interests, who wanted to maximise his own well-being and that of his wife and family, is also an obvious candidate. Scions of bourgeois Deventer families tended to take the boat over the rivers IJssel and Rhine up to Cologne in order to broaden their horizons by attending that town’s university.^78^ This academic orientation is reflected in folios 22 and 23, which explain in Latin how to prepare Cologne’s popular ship cookies and how to produce a type of jelly made of fish from the Rhine. The user of 345 would have been no stranger to the customs of Cologne, and the miscellany does indeed reflect the tastes and interests of someone who had spent some years at an *artes* faculty.

The obstetrical and gynaecological as well as culinary information also reveal that women were avid users of such information treasure troves.^79^ It is with this in mind that it is worth comparing Sloane 345 with a contemporary miscellany compiled by the heads of another type of household, the noblewomen Margaretha van Gymnich and Clara van Zollenhardt, which is still preserved at their family’s castle of Moyland in Anholt on the present Dutch-German border and some 30 miles from Deventer.^80^ The former directed a local physician, Arent Schryver, to copy and translate several treatises dealing with various topics: the distilling of spirits; the treatment of pestilence; venesection; and the use of herbs. Later on, Clara attached these treatises to her own collection of guidance literature, which comprised texts on gynaecology, an extensive medical handbook in Dutch combined with a long list of herbal remedies, and numerous technical, pharmaceutical and culinary recipes. She also ordered the transcription of two tables on how to calculate the date of Easter and how to determine the right dates for bloodletting. These directives were amply supplemented with information from other sources. In fact, the ladies owned a beautifully illustrated copy of Johannes Hartlieb’s *Herbal*, a prayer book opening with a prayer to Roche, the popular plague saint, and a prose Passion of Christ.^81^ It is highly probable that the Deventer owner of Sloane 345 also used his miscellany in combination with other information at hand in his library.

Sloane 345 has its origin in the hometown of the *Devotio Moderna* and thus might have been produced in the House of the Brethren, located in the city centre.^82^ This is in line with the fact that monasteries possessed such vademecums, too, which were to be consulted in conjunction with the religious teaching materials in their libraries.^83^ The Deventer Brethren maintained strong ties with the town’s leading circles and most of them had the advantage of a good education. The Brethren had sufficient money and writing materials, as well as the time and expertise, to produce such a comprehensive miscellany. Copying manuscripts, and particularly creating *florilegia* of various texts, was their core trade.^84^ Next to that, citizens who entered the religious communities of the Modern Devouts often took their own books with them, while their family members and friends also bestowed them with reading materials. As a result, their libraries were not only rich but diverse.^85^ Apart from devotional material, they held medical texts as well as other instructional works. The Deventer town councillor and diplomat Johan Marquard, for instance, donated a copy of John of Salisbury’s *Mirror for Princes* to the Heer Florens house, the leading foundation of the Modern Devouts. The town’s physician Reyner Oesterhusen donated medical handbooks to the same institution.^86^ Of course, in the tightly knit urban religious communities, people sometimes fell ill and were faced with a variety of well-being issues. Furthermore, the Brethren had to solve many household problems, varying as widely from how to clean their clothes and houses to how to prepare ink for the manuscripts they produced. Most importantly, city-dwellers regularly visited the houses to seek counsel from the Brethren, and in so doing they participated in their ‘knowledge community’.^87^ Sloane 345 exemplifies the kind of information they sought for their betterment.

An active assistance approach covering the same range of topics can already be seen in a somewhat older manuscript from the Frisian friar Bernard Rordahusim, who was connected to the houses of the Brethren in Münster and Hildesheim.^88^ The knowledge it provided in Latin, Middle-Dutch, Middle-German and Frisian was meant both for himself and for those who sought help from him. The guidelines oscillate between aid in arithmetic, health advice, guidance for following religious rules, and prescriptions for the preparation of stationery, pens, and ink, as well as information on the medicinal and culinary use of spices. The manuscript therefore displays the same helpful approach as Sloane 345. The persistent desire to improve the human condition, first on Earth and consequently in the afterlife, is aptly summarised by the repeatedly used slogan ‘*om te weten* – in order to know’. It wasn’t by chance that Bernard copied a world map in colour that had information on the winds and elements. Clearly, knowledge was viewed as a tool that could assist people in finding their place both on Earth and in life (f. 118v—Figure 4). Such orientation and usage is also confirmed by the research of Sara Ritchey, who found similar results when researching Flemish Cistercian nuns and beguines. They also compiled miscellanies that hinged on spiritual as well as physical well-being.^89^

**Fig. 4:**
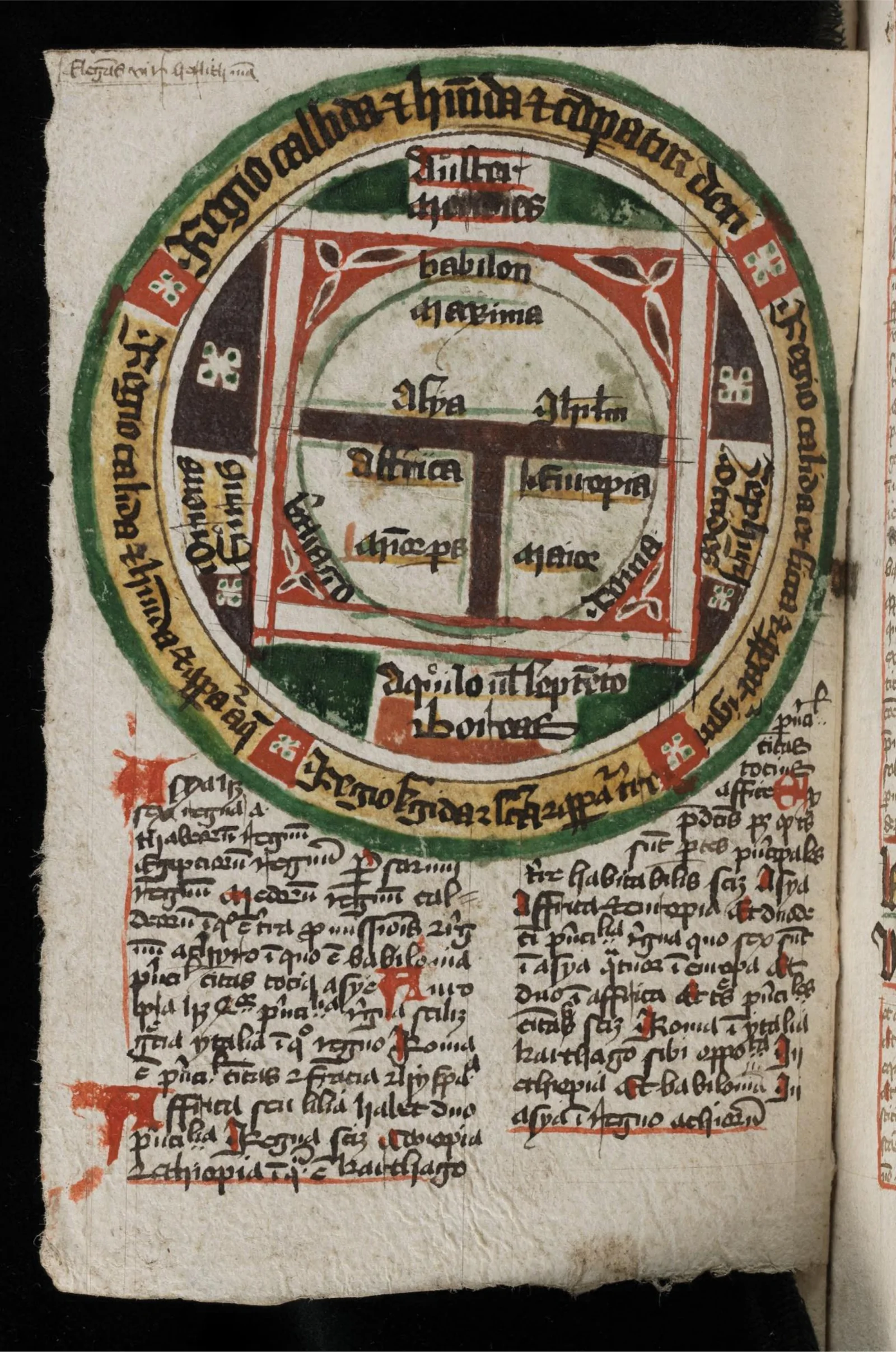
Worldmap with the characteristics of regions and winds. Basel, Universitätsbibliothek, F VII 12, f. 118v-s1v. Courtesy of University Library Basel, http://www.e-codices.ch/en/ubb/F-VII-0012/118v-s1v

To conclude, the multifaceted content of Sloane 345 must have found ready consumers in Deventer. For instance, the Modern Devouts’ first aim was to search for their inner self and to develop into better human beings, but this was impossible without being in good health. But this also holds true for those who sought their advice. Miscellanies were of equal usefulness across all ‘households’, no matter how we might define this term—a term which, along with religious communities, would have referred just as much to the inhabitants of castles as it did to those of bourgeois townhouses. At the time, people who put together such miscellanies combined them with other handwritten and printed books and may have built reference libraries that covered the whole gamut of knowledge on well-being. All participants in the ‘knowledge communities’ they constituted were engaged in the creation and maintenance of a good life for themselves and others.

## Conclusion

The Deventer humanist and rector of the city’s Latin School, Alexander Hegius (ca. 1435–1498), once composed a poem on the Aristotelian ‘Golden Mean’. No doubt his students were obliged to translate his poetic interpretation of Aristotle’s *Nicomachean Ethics* and learn it by heart (Figure 5).^90^ The warnings of the poem—do not drink too much, and do not indulge in the delights of Venus but restrict yourself to a life of moderation and become an esteemed citizen—complement the practical contents of the Deventer miscellany. Both are suffused with the morality of the *vita activa*, hinging on applicability, utility and expertise, steering oneself towards common profit and betterment. All of this is rooted in the late medieval household. Moreover, the composition of the miscellany indicates that the medieval ideal of measure was not restricted to health and medicine but encompassed all aspects of life.^91^

**Fig. 5:**
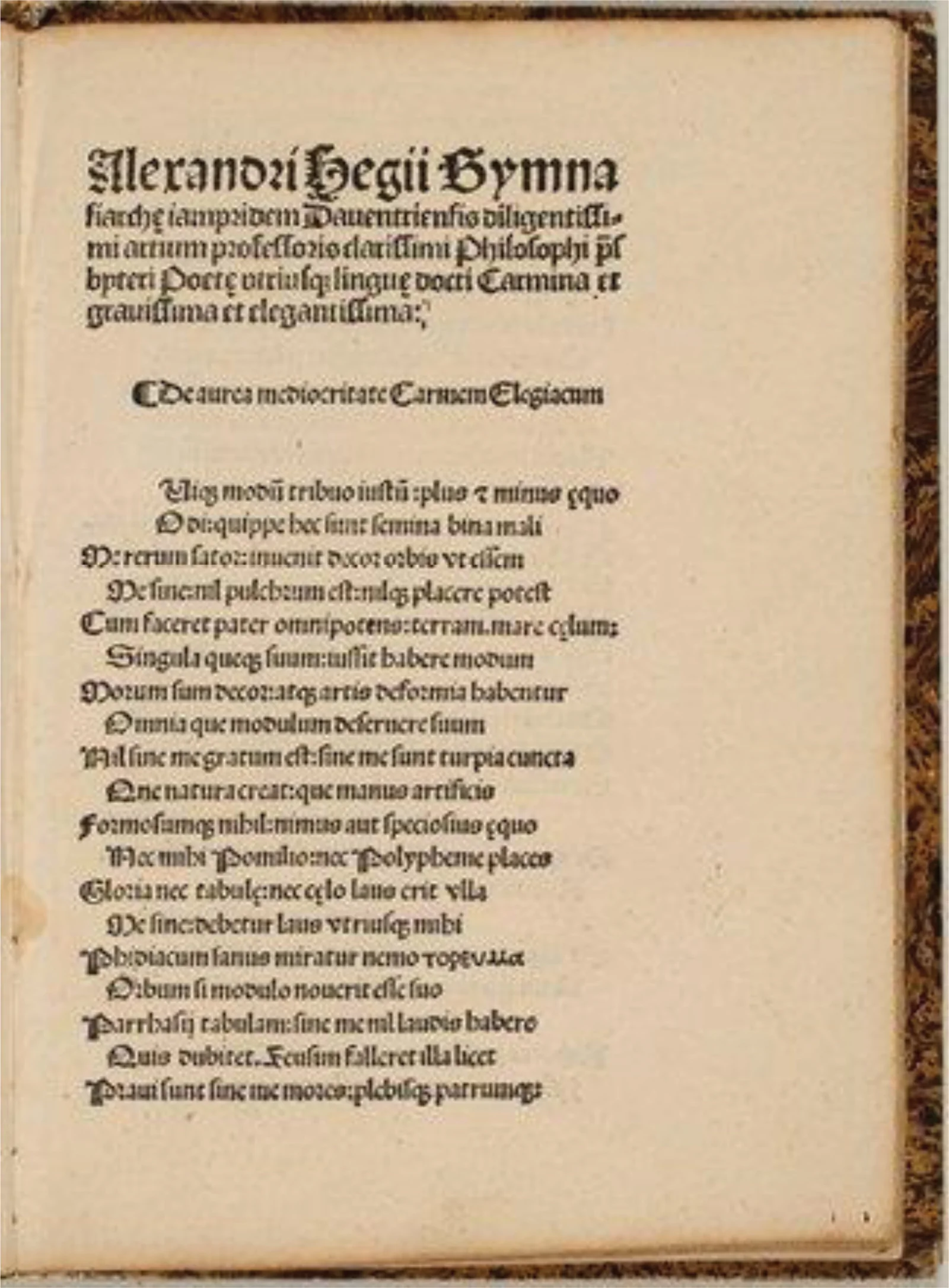
Poem on the Golden Mean by Alexander Hegius. Deventer: Richard Pafraet, ca. 1503, Courtesy of Royal Library, the Hague.

Understanding the role of such a pursuit of ‘betterment’ is consistent with the medieval model of health, which was to all intents and purposes a comprehensive guide to a just life. *Sanitas* (health) was often put on a par with *valetudo* (health condition) and *salubritas* (well-being), implying that the task of medical doctors was not only *conservatio sanitatis* (the preservation of health) but also *perfectio vitae* (the perfection of life). The adjective for healthy, *salus*, could also refer to spiritual welfare and, ultimately, salvation. Moreover, medieval medicine was based on moderation and balance, as found in the lifestyle medicine of Sloane 345.^92^ Precisely along these lines, throughout the Middle Ages men and women made constant efforts to better themselves and their lives. Their ultimate goal was to become experts in a Christian way of life and to improve their condition, which called for mastery of vital skills—what I have referred to as a ‘culture of expertise’. By the end of the Middle Ages, this attitude had assumed an increasingly functional character, which can be seen especially in the various urban miscellanies. It is therefore timely to study them within the context of a wider and more comprehensive betterment culture. Knowledge was considered indispensable, because it transformed its users into accomplished citizens and good Christians.

With this in mind, in the context of the present study of this Deventer specimen of the ‘whole book’, I propose to distance the medieval miscellany from the recent focus on domesticity and its primary association with women, the home, the homely and the family.^93^ In my opinion, the miscellany’s purpose was driven by the desire to equip oneself with the knowledge to create well-being; in consequence, it was primarily a tool for achieving a better life. My in-depth analysis of Sloane 345 also suggests that the arguments of Riddy and Harry on the coming into being of a ‘burgeis identity’ and a ‘language of common profit’ during the medieval period match the content and goals of the collection—and by extension the advice text or secret book in general—more closely than the explorations of domesticity offered in a narrower sense by Demaitre, Kowaleski and French, although this is partly a matter of definition.^94^ The users likely participated in a household that was tied together by co-residents, employees and visitors who shared various activities and counselled each other, but did not necessarily constitute a (nuclear) family with an accompanying domestic mentality. Thus, it seems more useful to reformulate ‘domestic expressions’ into ‘signs of communally shared approaches and attitudes with the aim of betterment’.

In the wake of recent scholarly work on ‘secret’ literature and recipe collections in connection with the how and why of the dissemination of knowledge, centred on the investigation of England and Italy, it is time to start a Europe-wide study of practical texts in manuscript and print following the example of the exciting EMROC project on Early Modern Cooking Recipes, but extending this to the Late Middle Ages and especially to include medieval miscellanies of the type of Sloane 345 as a forerunner of the later advice and ‘secret’ books.^95^ Only on the basis of such an effort can we genuinely establish whether or not the urge for betterment, with possibly even the pursuit of universal human traits, was a Europe-wide phenomenon, and what the regional and sociocultural varieties might have been.^96^ At the same time, this would allow us to respond to recent pleas to take a more flexible view of the ‘vernacularization’ of the Middle Ages, i.e. not as a unilateral transformation from Latin to the European vernacular but as evidence of the growing accessibility and popularity of academic, medical and theological texts amongst those who were not accustomed to reading them, but who were interested in their own overall well-being.^97^

This comprehensive analysis of Sloane 345 has unearthed at least one common propensity: to proactively ‘better’ both the individual and collective life; equally, the study has revealed various ways of life based on the active attainment of expertise which started in the late Middle Ages and ended around the birth of the Modern era. This supports the findings of Leong and Rankin, who have explored the resurgence of interest in historical know-how in their study of the topic of drug testing, while pointing to a significant degree of continuity from the (late) Middle Ages to the eighteenth century.^98^ The habitus that followed increasingly relied on personal experience, which merged text-based knowledge of past practices with hands-on experience. Hence, it is time to truly examine the experiential learning of historical readers, as revealed in medieval miscellanies like Sloane 345. Taking seriously their practicality and their capacity to cope with long-term questions of life and death, whose primary focus remained on the creation of balance and a Good Life, puts our current technological and digital health utopias into perspective.^99^

## Epilogue: Hans Sloane and His Miscellany

The miscellany under scrutiny in the above article was bequeathed to the British Library by Sir Hans Sloane (1660–1753), a society medical doctor who practised in London. His immense wealth hailed from the commercial sale of medications, which allowed him to indulge in his hobby of buying huge numbers of medical manuscripts and early books, as well as other curiosities.^100^ The history of the Middle Ages is extensively represented in his collection, which comprised papal bulls, eternal calendars, block books, seals, gems and religious objects.^101^ In fact, the collecting of medieval manuscripts was highly fashionable in seventeenth- and eighteenth-century London. Sloane’s pursuits aligned with the contemporary passion for the continental, specifically Dutch, science.^102^ His correspondence with the Leiden professor of medicine Herman Boerhaave (1668–1738) is a reflection of this, as the latter was also a dedicated fan of medieval manuscripts.^103^

By spending a fortune on medieval manuscripts, Sloane acknowledged the significance of medical texts from the past for contemporary health care. The construction of his library was thus not merely driven by bibliophile taste, a fact which can be gathered from his collecting strategy, which was based on the medical-historical bibliography composed by the Dutch anatomist and librarian of the University of Franeker J.A. van der Linden (1609–1664).^104^ The updated version, *Lindenius Renovatus*, served as a catalogue for Sloane’s library. Its annotations reveal how highly Sloane valued the practices of his professional predecessors. To guarantee maximum profit from the expertise of centuries of medical practitioners, he chose to buy annotated texts that provided additional useful information.

The 1745 *Account of a Most Efficacious Medicine for Soreness, Weakness, and Several Other Distempers of the Eyes* is Sloane’s only published medical work. By arguing that cures invented ages ago are likely to still be effective because they have already been tested and improved by patients and their doctors, the pamphlet demonstrates the potential of historical investigation for the advancement of medical science. In fact, many medical therapies lay waiting in the manuscripts for rediscovery.^105^ Sloane’s interest in historical ophthalmology is further reflected in his collections, as he cherished an impression in brass of the seal of the Parisian hospital of the Quinze Vingts for the blind, founded by the medieval French king and saint Louis IX.^106^ Ergo, the history of medicine in its broadest sense was considered fundamental for further progress of the medical discipline.

Hans Sloane’s ownership of the Deventer miscellany and many comparable works reveals a second blossoming of a ‘culture of expertise’ in the Enlightenment. Such an observation taps into the findings of Nancy Siraisi and Gianna Pomata on the early modern use of *historia* in medicine and natural philosophy.^107^ These disciplines shared a ‘fundamentally empirical orientation with a strong commitment to ancient texts as models and practical guides’.^108^ To become an expert in betterment, Sloane purchased as well as avidly studied medieval advice miscellanies that were storehouses of information on the topic and led him to proven remedies. In sum, his medical interests confirm that at the time intellectuals saw reason and empiricism as a pair grounded in the transference and adjustment of earlier knowledge. All these contentions lead to the historiographical stance that after the mechanistic phase of the seventeenth century, attention to living nature returned. Natural philosophers started to investigate how human interactions changed over time, and thus did not separate the natural and humanistic sciences: nature became historised.^109^

## Footnotes

1 M. A. E. Nickson, ‘Books and Manuscripts’, in Arthur MacGregor, ed., *Sir Hans Sloane: Collector, Scientist, Antiquary, Founding Father of the British Museum* (London: British Museum Press, 1994), 263–277 and Arnold Hunt, ‘Sloane and a Collector of Manuscripts’, in A. Walker, ed., *From Books to Bezoars. Sir Hans Sloane and His Collections* (London: British Library, 2012), 190–206.

2 Description and contents: This article benefits greatly from: Anja Groeneveld, *Het medisch compendium van Reyner Oesterhusen. Onderzoek naar het handschrift Sloane 345* (unpublished master’s thesis, University of Groningen, 2015).

3 Erwin Huizenga, *Een Nuttelike Practijke van Cirurgien: Geneeskunde en Astrologie in het Middelnederlandse Handschrift Wenen, Österreichische Nationalbibliothek, 2818* (Hilversum: Verloren, 1997), 81, 88, 105, 193, 405, 413. The prescriptions and recipes are edited by W. Braekman, *Medische en Technische Middelnederlandse Recepten: Een tweede bijdrage tot de geschiedenis van de vakliteratuur in de Nederlanden* (Gent: Koninklijke Vlaamsche Academie, 1975), 1218–1220.

4 Ria Jansen-Sieben, *Repertorium van de Middelnederlandse Artes-Literatuur* (Utrecht: HES, 1989). Also available from: Brill Leiden https://doi-org.proxy-ub.rug.nl/10.1163/9789004619593.

5 M. A. E. Nickson, ‘Hans Sloane, Book Collector and Cataloguer, 1682-1698’, *The British Library Journal*, 1988, 14, 52–89; Nickson, ‘Books and Manuscripts’ and Hunt, ‘Sloane Collector’, 190–207.

6 Introductions: Elaine Leong and Alisha Rankin, eds, *Secrets and Knowledge in Medicine and Science, 1500-1800* (Farnham: Ashgate, 2011); Alisha Rankin, *Panaceia’s Daughters: Noblewomen as Healers in Early Modern Germany* (Chicago: University of Chicago Press, 2013); and Sandra Cavallo and Tessa Story, eds, *Conserving Health in Early Modern Culture: Bodies and Environments in Italy and England* (Manchester: Manchester University Press, 2017).

7 Sandra Cavallo, ‘Early Vernacular Medical Advice Books and Their Popular Appeal in Early Modern Italy’, *Nuncius*, 2021, 36.2, 289 and 300–301.

8 Compare: Sabrina Minuzzi, ‘15th-Century Practical Medicine in Print: Beyond the Profession, towards the Miscere Utile Dulci’, *Nuncius* 2021, 36.2, 199–263 and Natasha Glaisyer and Sara Pennell, ‘Introduction’, in Natasha Glaisyer and Sara Pennell, eds, *Didactic Literature in England 1500-1800: Expertise Constructed* (Oxford: Taylor & Francis Group, 2003), 1–18.

9 Luke Demaitre, ‘Domesticity in Middle Dutch “Secrets of Men and Women”’, *Social History of Medicine*, 2001, 14, 1–25.

10 Katherine L. French, *Household Goods and Good Households in Late Medieval London: Consumption and Domesticity After the Plague* (Philadelphia: University of Pennsylvania Press, 2021), Introduction and 217–218.

11 The propagation of the good life in its medieval guise is demonstrated by Steven J. Jensen, *Living the Good Life* (Washington, DC: Catholic University of America Press, 2013).

12 This article speaks of betterment to avoid the discussion on Western dynamics proposed by Paul Slack, *The Invention of Improvement: Information and Material Progress in Seventeenth-Century England* (Oxford: Oxford University Press, 2015).

13 Felicity Riddy, ‘“Burgeis” Domesticity in Late-medieval England’, in Maryanne Kowaleski and P. J. P. Goldberg, eds, *Medieval Domesticity. Home, Housing and Household in Medieval England* (Cambridge: Cambridge University Press, 2008), 14–36, esp. 20, 30.

14 David Harry, *Constructing a Civic Community in Late Medieval London: The Common Profit, Charity and Commemoration* (Suffolk: Boydell Press, 2019), esp. Ch. 6.

15 William Eamon, ‘How to Read a Book of Secrets’, in Elaine Leong and Alisha Rankin, eds, *Secrets and Knowledge in Medicine and Science, 1500-1800* (Farnham: Ashgate 2011), 23–46, esp. 30 and 44. The definition is slightly adapted. I suggest broadening it to include the consumers of this instruction material. On ‘secrets’, see also Allison Kavey, *Books of Secrets: Natural Philosophy in England, 1550-1600* (Urbana: University of Illinois Press, 2007) and Leong and Rankin, ‘Introduction’, *Secrets and Knowledge*, 1–19.

16 Glaisyer and Pennell, *Didactic Literature*.

17 See the above quoted literature. Also Minuzzi, ‘15th-Century Practical Medicine’, which is entirely on Italy, and Cavallo and Storey, *Conserving Health.*

18 Pamela H. Smith, ‘What Is a Secret? Secrets and Craft Knowledge in Early Modern Europe’, in Elaine Leong and Alisha Rankin, eds, *Secrets and Knowledge in Medicine and Science, 1500-1800*, 47–66 and Katherine Park, *Secrets of Women: Gender, Generation, and the Origins of Human Dissection* (New York: Zone Books, 2010).

19 Leong and Rankin, *Secrets and Knowledge*, 3.

20 Cavallo, ‘Medical Advice Books’.

21 Stephen G. Nichols and Siegfried Wenzel, *The Whole Book: Cultural Perspectives on the Medieval Miscellany* (Ann Arbor: University of Michigan Press, 1996) and Karen Pratt and Hannah Morcos, eds, *The Dynamics of the Medieval Manuscript: Text Collections from a European Perspective* (Göttingen: V&R Academic, 2017). The survey of terminology is by Marilena Maniaci, ‘Miscellaneous Reflections on the Complexity of Medieval Manuscripts’, in S. Corbellini, ed., *Collecting, Organizing and Transmitting Knowledge: Miscellanies in Late Medieval Europe*. *Bibliologia*, vol. 49 (Turnhout: Brepols, 2018), 12–22.

22 A source of inspiration for this article is: Susanna Fein, ed., *Interpreting MS Digby 86: A Trilingual Book from Thirteenth-Century Worcestershire of Manuscript Culture in the British Isles* (Martlesham: Boydell & Brewer, 2019), esp. Marjorie Harrington, ‘Science, Medicine, Prognostication: MS Digby 86 as a Household Almanac’, 51–72.

23 Nikolaus Staubach, ‘*Diversa raptim undique collecta*: Das Rapiarium im geistlichen Reformprogramm der Devotio moderna’, in K. Elm, ed., *Florilegien, Kompilationen, Kollektionen. Literarische Formen des Mittelalters* (Wiesbaden: Harrassowitz, 2000), 115–148.

24 Josef Arlinghaus et al., ed., *Transforming the Medieval World: Uses of Pragmatic Literacy in the Middle Ages* (Turnhout: Brepols, 2006).

25 Nichols and Wenzel, *The Whole Book* and Derek Pearsall, ‘The Whole Book: Late Medieval English Manuscript Miscellanies and Their Modern Interpreters’, in Stephen Kelly and John J. Thompson, eds, *Imagining the Book* (Turnhout: Brepols, 2005), 17–29. Also, Fein, ‘Interpreting Ms Digby 86’.

26 Nickson, ‘Books and Manuscripts’, 269 and Plate 36.

27
http://www.bl.uk/manuscripts/FullDisplay.aspx?ref=Sloane_MS_2435.

28 Rudolph M. Bell, *How to Do It. Guides to Good Living for Renaissance Italians* (Chicago: Chicago University Press, 1999); Sandra Cavallo and Tessa Storey, *Healthy Living in Late Renaissance Italy* (Oxford: Oxford University Press, 2014) and Cavallo and Storey, *Conserving Health*. Compare: Slack, *Improvement,* who concentrates on the seventeenth century.

29 Reflections on the how and why of these books is given by: Jennifer Richards, ‘Useful Books: Reading Vernacular Regimens in Sixteenth-Century England’, *Journal of the History of Ideas*, 2012, 73, 247–271; Elaine Leong, ‘Collecting Knowledge for the Family: Recipes, Gender and Practical Knowledge in the Early Modern English Household’, *Centaurus*, 2013, 55, 81–103 and Melissa Reynolds, ‘“Here Is a Good Boke to Lerne”: Practical Books, the Coming of the Press, and the Search for Knowledge, ca. 1400-1560’, *Journal of British Studies* 2019, 58, 259–288.

30 Arlinghaus, *Pragmatic Literacy*.

31 Compare, Minuzzi, ‘15th-Century Practical Medicine’.

32 Classical introductions: Herbert Grabes*, Speculum, Mirror und Looking-Glass: Kontinuität und Originalität der Spiegelmetapher* (Tübingen: Max Niemeyer Verlag, 1973) and Marilyn Nicoud, *Les Régimes de Santé au Moyen Âge: Naissance et diffusion d’une écriture médicale en Italie et en France (XIIIe- XVe Siècle)* (Rome: École Française de Rome, 2013).

33 Lynette Hunter, John Barnard, D.F. McKenzie and Maureen Bell, ‘Books for Daily Life. Household, Husbandry, Behaviour’ in John Barnard, ed, *The Cambridge History of the Book in Britain* (Cambridge: Cambridge University Press, 2002), 514–532.

34 George Keiser, ‘Practical Books for the Gentleman’, in Lotte Hellinga and J. B. Trapp, eds, *The Cambridge History of the Book in Britain* (Cambridge: Cambridge University Press, 1999), 470–474 on ‘voracious appetite for writings of educational edification’.

35 Leong and Rankin, *Secrets and Knowledge* and Cavallo, ‘Medical Advice Books’.

36 Wolffram Schmitt, *Deutsche Fachprosa des Mittelalters* (Tübingen: De Gruyter, 1972); Ria Jansen-Sieben, ‘Middelnederlandse vakliteratuur’, in Gundolf Keil and Peter Assion, eds, *Fachprosaforschung. Acht Vorträge zur mittelalterlichen Artesliteratur* (Berlin: Schmidt, 1974), 24–69; and E. Huizenga, O. Lie and L. Veltman, eds, *Een wereld van kennis. Bloemlezing uit de Middelnederlandse artesliteratuur* (Hilversum: Verloren, 2002). Jansen-Sieben, *Repertorium*, defines artes-texts as writings which all have a utilitarian, instructive and not mainly recreational, aesthetic, religious or emotional goal, XXII.

37 Jansen-Sieben, *Repertorium*; Huizenga, *Nuttelike Practijke*; and Orlanda S. H. Lie and Joris Reynaert, eds, *Artes in Context: Opstellen over het handschriftelijke milieu van Middelnederlandse Artesteksten* (Hilversum: Verloren, 2004).

38
http://www.bl.uk/manuscripts/FullDisplay.aspx?ref=Sloane_ms_345. See also the blog by Chantry Westwell, https://blogs.bl.uk/digitisedmanuscripts/2015/04/ointments-and-potions.html, accessed 11 November 2022.

39 Henk Slechte, *Geschiedenis van Deventer. Oorsprong en Middeleeuwen* (Zutphen: Walburg Pers, 2010), 144.

40 S. B. J. Zilverberg, *David van Bourgondië, Bisschop van Terwaan en van Utrecht (1427-1496)* (Groningen-Jakarta: J.B. Wolters, 1951), 88.

41 Incipit: *Johannes behemens de institutione medici. O tu corporis curam gerens per omnia auxiliari*. See Bibliotheca Vaticana MS. 2418, fol. 209r and Lynn Thorndike and Pearl Kibre, *A Catalogue of Incipits of Mediaeval Scientific Writings* (London: Mediaeval Academy of America, 1963), 973.

42 Michael Mc Vaugh, ‘Arnald of Villanova’, in Noretta Koertge ed., *Complete Dictionary of Scientific Biography* (Detroit, Mich.: Charles Scribner’s Sons, 2008), I, 289–291.

43 See John Ordronaux, *Regimen sanitatis salernitanum. Code of Health of the School of Salernum* (Philadelphia: J.B. Lippincott, 1970), who includes the texts that accompanied the Regimen of Salerno.

44 Brian Lawn, *The Salernitan Questions: An Introduction to the History of Medieval and Renaissance Problem Literature* (Oxford: Clarendon Press, 1963), 99.

45 J. de Ketham, *The Fasciculus Medicinae of Johannes de Ketham, Alemanus: Facsimile of the First (Venetian) Edition of 1491*, with an English translation by Luke Demaitre and commentary by Karl Sudhoff; translated and adapted by Charles Singer (Birmingham, AL: The Classics of Medicine Library, 1988).

46 On the dissemination of this text: Erwin Huizenga, *Tussen Autoriteit en Empirie: De Middelnederlandse chirurgieën in de veertiende en vijftiende Eeuw en hun maatschappelijke context* (Hilversum: Verloren, 2003).

47 The bump: Braekman, *Middelnederlandse Recepten*, nr 1175.

48 Demaitre, ‘Secrets’.

49 On this in general: Park, *Secrets of Women*.

50 Monica Green corrected the title; see her 2015 reaction in https://blogs.bl.uk/digitisedmanuscripts/2015/04/ointments-and-potions.html. Accessed 11 November 2022.

51 Park, *Secrets of Women.*

52 Braekman, *Middelnederlandse recepten*, nrs 738, 751, 756.

53 For instance: Braekman, *Middelnederlandse recepten*, nrs 716 and 770 on ruining the embryo. Compare: J. M. Riddle, ‘Oral Contraceptives and Early-Term Abortifacients during Classical Antiquity and the Middle Ages’, *Past & Present*, 1992, 132, 3–32.

54 Braekman, *Middelnederlandse recepten,* nrs 890–908.

55
*Ibid*., nr 627.

56 Sheila Delany, ‘“Mulier est hominis confusio”: Chaucer’s Anti-popular Nun’s Priest’s Tale’, *Mosaic: A Journal for the Interdisciplinary Study of Literature*, 1984, 17, 1–8.

57 Van Schoonenberghe, ‘Genever (Gin): A Spirit Drink Full of History, Science and Technology’, *Sartonia*, 1999, *1*, 100, 112.

58 Braekman, *Middelnederlandse recepten*, nrs 590–626.

59 Edition: M. M. van Dantzig, ‘Een vijftiende-eeuwsch receptenboek’, *Oud Holland. Journal for the Art of the Low Countries*, 1936, 53, 207–218.

60 S. Folkerts, ed., *Deventer Boekenstad* (Deventer: Walburg Pers, 2020).

61 Braekman, *Middelnederlandse recepten*, nrs 612, 613, 623.

62
*Ibid.*, nrs 629, 630.

63 Compare: Monserrat Cabré, ‘Beautiful Bodies’, in Linda Kalof, ed., *The Cultural History of the Body in the Medieval Age* (London: Bloomsbury, 2010), 121–140.

64 Braekman, *Middelnederlandse recepten*, nrs 711, 712.

65
*Ibid*., nrs 668 and 712.

66
*Ibid*., nr 1052.

67
*Ibid*., nrs 735, 737, 774, 775 and 1066 (lethargy).

68
*Ibid*., nr 511.

69 Lawn, *Salernitan Questions*, 99.

70 David Eugene Smith, George Plimpton, and Augustus de Morgan, *Rara Arithmetica: A Catalogue of the Arithmetics Written Before the Year MDCI*, 4th edn (New York: Chelsea Publications, 1970), 70, 73.

71 Huizenga, ‘Cutting and Writing’.

72 This is counted by Jansen-Sieben in her catalogue, *Repertorium*, XIV.

73
*Catalogus Librorum Manuscriptorum Bibliothecae Sloanianae (Manuscripts 1-1091)* (London: British Museum, no date), 56.

74 J. van Herwaarden, ‘Medici in de Nederlandse samenleving in de late middeleeuwen (14e-16e eeuw)’, *Tijdschrift voor Geschiedenis*, 1983, 96, 362–364.

75 Hypothesis of Groeneveld, *Sloane 345*.

76 The situation was probably not very different from that in Italian cities: see James Shaw and Evelyn S. Welch, *Making and Marketing Medicine in Renaissance Florence* (Amsterdam: Rodopi, 2011), 42–45, 295–296.

77 Minuzzi, ‘15th-Century Practical Medicine’, 214–215.

78 J. F. Benders, *Bestuursstructuur en schriftcultuur: een analyse van de bestuurlijke verschriftelijking in Deventer tot het eind van de vijftiende eeuw* (Kampen: IJsselacademie, 2004) on slender ties with Cologne, 126, 298, 323–326.

79 This is also found by: Sara Margaret Ritchey and Sharon T. Strocchia, eds, *Gender, Health, and Healing, 1250-1550* (Amsterdam: Amsterdam University Press, 2020).

80 Anholt Fürstlich Salm-Salm’sche Bibliotheek (hereafter FSSB), Hs. 33. On this: Annick Kothuis ‘*Uit hoofde van vriendschap’ Een medisch verzamelhandschrift, twee adellijke dames en een arts in de vijftiende eeuw* (unpublished master’s thesis, Groningen, 2013).

81 FSSB, ‘Prayer book’, Hs. 40; FSSB, ‘Die Passie ons leven heren Jhesu Christi’, Hs. 39; FSSB, ‘Anholter-Moyländer Kräuterbuch’, Hs. 46. On the latter: Irmgard Müller and Michael Martin, ‘Die Handschrift ‘Ms. 46’ der Fürstlich Salm-Salm’schen Bibliothek der Wasserburg Anholt. Kräuterbuch des Johannes Hartliebs’, *Wissenschaftlicher Begleitband zur Faksimile-Ausgabe* (Bedburg-Hau: Museum Moyland, 2004), 9–15.

82 John van Engen, *Sisters and Brothers of the Common Life. The Devotia Moderna and the World of the Later Middle Ages* (Philadelphia: University of Pennsylvania Press, 2008).

83 For instance: Jansen-Sieben, *Repertorium*, 231 (Walters Art Gallery Ms 171 and 333–334 and Royal Library, The Hague, KAW XXVIII, both owned by religious women). Compare: Sara Margaret Ritchey, *Acts of Care: Recovering Women in Late Medieval Health* (New York: Cornell University Press, 2021).

84 Thomas Kock, *Die Buchkultur der Devotio Moderna. Handschriftenproduktion, Literaturversorgung und Bibliotheksaufbau im Zeitalter des Medienwechsels* (Frankfurt am Main: Peter Lang, 1999).

85 M. E. Kronenberg, ‘De Bibliotheek van Het Heer-Florenshuis te Deventer’, *Nederlands Archief voor Kerkgeschiedenis / Dutch Review of Church History*, 1916, 9.1, 150–164 and 252–300, esp. 255–256.

86 From the Heer Florenshuis, now in Deventer Atheneum Bibliotheek: HS 106 Bertrucius Bononiensis, *Lectorium Compendium artis medicae* and HS 109 *Clarificationes morborum JohannIs de Tornamira decani praeclari studij*. HS 108 is the *Lilium Medicinae* by Bernard de Gordon.

87 Pieter Boonstra*, Reading by Example. The Communication of Religious Knowledge in the Collationes of the Brothers of Common Life* (PhD thesis, University of Groningen, 2021).

88 Basel, University Library, F III-0012. Description and digitalized manuscript: https://www.e-codices.unifr.ch/en/description/ubb/F-VII-0012/HAN.

89 Ritchey, *Acts of Care*.

90 J. C. Bedaux, *Hegius Poeta: Het leven en de Latijnse gedichten van Alexander Hegius* (Deventer: Salland de Lange, 1998), 86–87.

91 Joel Kaye, *A History of Balance, 1250-1375: The Emergence of a New Model of Equilibrium and Its Impact on Thought* (Cambridge: Cambridge University Press, 2014).

92 H. Schipperges, ‘Gesundheit’, in R. Auty ed., *Lexikon des Mittelalters* (Stuttgart: Metzler, 1989), 4, 1412–1413 and Faith Wallis, ‘Isidore of Seville and Science’, in Andrew Fear and Jamie Wood, *A Companion to Isidore of Seville* (Leiden: Brill, 2019), 182–221. Also, Kaye, *History of Balance*, esp. 128–240.

93 Demaitre, ‘Domesticity’; Kowaleski and Goldberg, *Medieval Domesticity*, 1–13; and French, *Household Goods*.

94 Riddy, ‘“Burgeis” Domesticity’ and Harry, *Civic Community*.

95 Leong and Rankin, *Secrets*, passim. On EMROC: https://emroc.hypotheses.org/. Special issue ‘Celebrating Ten Years of the Early Modern Recipe Online Collective’ of *Early Modern Studies Journal*, 2022, *8.*

96 Jeff Steele, ‘Happiness’, in Thomas Williams, ed., *The Cambridge Companion to Medieval Ethics* (Cambridge: Cambridge University Press, 2018), 127–149.

97 Cavallo, ‘Medical Advice Books’, 265–266.

98 Elaine Leong and Alisha Rankin, ‘Testing Drugs and Trying Cures: Experiment and Medicine in Medieval and Early Modern Europe’, *Bulletin of the History of Medicine*, 2017, 91, 157–182.

99 Robert Peckham, ‘A Health Emergency Is No Time to Side-line the Medical Humanities’, *Times Higher Education Supplement*, 2020, *2446*.

100 MacGregor, *Sir Hans Sloane* and Walker, *From Books to Bezoars*.

101 John Cherry, ‘Medieval and Later Antiquities’, in A. MacGregored., *Sir Hans Sloane: Collector, Scientist, Antiquary, Founding Father of the British Museum* (London: British Museum Press, 1994), 198–221.

102 Hunt, ‘Sloane Collector’, 197 and 202.

103 H. G. M. Jorink, ‘Sloane and the Dutch Connection’, in A. Walker ed., *From Books to Bezoars. Sir Hans Sloane and His Collections* (London: British Library, 2012), 57–70.

104 J. A. van der Linden, *De medici libri duo scriptis* (Amsterdam; Ioannes Blaeu, 1637, 1651, 1662).

105 Hunt, ‘Sloane Collector’, 203.

106 Cherry, ‘Antiquities’, 212–213, esp. note 75.

107 Gianna Pomata and Nancy G. Siraisi, eds, *Historia: Empiricism and Erudition* (Cambridge, Mass.: Chicago Press, 2011).

108 Nancy G. Siraisi, *History, Medicine, and the Traditions of Renaissance Learning* (Ann Arbor: Michigan University Press, 2007), 262.

109 Peter H. Reill, *Vitalizing Nature in the Enlightenment* (Berkeley, University of California Press, 2005), 1–15.

## Notes

Catrien Santing is a medievalist specialising in the Cultural History, Medical History and Body History of the Late Middle Ages. She has published on the cultural denotation of body parts, especially the heart, and late medieval intellectual history. Currently, she is finishing a book on medieval lifestyle medicine in relation to the Good Life.

